# Evaluation of the Therapeutic Potential of *Spirulina* Polysaccharides on Carbon Tetrachloride-Induced Liver Fibrosis in Mice: A Study Based on the Interaction Between Gut Microbiota and Metabolites

**DOI:** 10.3390/nu18142215

**Published:** 2026-07-08

**Authors:** Min Li, Songyao Xu, Meiting Zhang, Xin Wang, Siyan Wang, Xinle Wang, Ruiping Hu, Huiting Xue

**Affiliations:** 1 Tumor Multimodal Digital and Intelligent Precision Diagnosis Engineering Research Center, School of Basic Medical Sciences, Inner Mongolia Medical University, Hohhot 010030, China; lm372925@163.com (M.L.); w1037419140@163.com (S.W.); 2 Molecular Biology Research Center, School of Basic Medical Sciences, Inner Mongolia Medical University, Hohhot 010030, China; maomiyaoyao@126.com (S.X.); swuzhang2025@163.com (M.Z.); 17547441138@163.com (X.W.); wxl020328@163.com (X.W.)

**Keywords:** liver fibrosis, *Spirulina platensis*, polysaccharides, intestinal flora, short-chain fatty acids

## Abstract

**Introduction**: Hepatic fibrosis represents a critical intermediate stage in the progression from chronic liver disease to cirrhosis and ultimately hepatocellular carcinoma. *Spirulina platensis*, a microorganism rich in bioactive compounds, contains several functional components, among which are *Spirulina* polysaccharides and phycocyanin. Both have been demonstrated to exhibit multiple biological activities, including antioxidant, anti-inflammatory, and immunomodulatory effects. However, the intervention effects and mechanisms of *Spirulina* polysaccharides and phycocyanin on CCl_4_-induced hepatic fibrosis remain unclear. 16S rRNA sequencing and non-targeted metabolomics technologies are employed to analyze bacterial taxa and metabolic pathways. **Methods**: Hepatic fibrosis (HF) was induced in male C57BL/6J mice via intraperitoneal injection of CCl_4_. After 1 week of modeling, mice were intragastrically administered SPP or PC for 4 consecutive weeks. Gut microbiota composition and fecal metabolites were analyzed using 16S rRNA gene sequencing and metabolomics. **Results**: SPP showed more pronounced protective effects than PC under the experimental conditions used in this study. SPP treatment significantly alleviated hepatic fibrosis in mice. Compared with the model group, SPP administration markedly increased the abundance of bacterial taxa and modulated fecal metabolic profiles, including short-chain fatty acid metabolism. **Conclusions**: SPP treatment mitigates CCl_4_-induced hepatic fibrosis in mice. These protective effects may be associated with the modulation of gut microbiota composition and fecal metabolic pathways, including those related to short-chain fatty acid metabolism.

## 1. Introduction

Hepatic fibrosis is a critical intermediate stage in the progression of chronic liver disease to cirrhosis and hepatocellular carcinoma. A key pathological feature is the activation of hepatic stellate cells (HSCs) following hepatocyte injury, accompanied by excessive deposition of extracellular matrix components, particularly collagen, leading to hepatic tissue remodeling and dysfunction [1,2]. Globally, the prevalence of non-alcoholic fatty liver disease (NAFLD)-associated hepatic fibrosis is increasing annually. A classic strategy for establishing hepatic fibrosis models in vivo combines high-fat diet-induced lipid metabolism disorders with exposure to chemical toxins such as carbon tetrachloride (CCl_4_) [3,4]. Free radicals generated from hepatic metabolism of CCl_4_ directly damage hepatocyte membranes, while a high-fat environment aggravates hepatic lipid accumulation; these two factors synergistically activate inflammatory signaling pathways and accelerate fibrogenesis, with pathological features closely resembling human metabolism-related hepatic fibrosis [5,6].

At present, no specific anti-fibrotic drugs are available for hepatic fibrosis in clinical practice. Existing interventions mainly focus on etiological control (e.g., lipid lowering, alcohol abstinence) and are insufficient to reverse established extracellular matrix deposition and fibrosis [7,8]. Exploring anti-fibrotic functional ingredients from natural biological resources has attracted increasing research interest in recent years. *Spirulina platensis*, a cyanobacterium rich in polysaccharides, phycocyanin, and other bioactive substances, is widely distributed in arid and semi-arid regions such as Inner Mongolia. Its major components include *Spirulina* polysaccharide (SPP) and phycocyanin (PC). However, studies on the intervention effects and mechanisms of *Spirulina platensis*-derived bioactive components—especially SPP and PC—on CCl_4_-induced hepatic fibrosis remain limited. Their differential effects on the gut–liver axis, fibrosis-related gene expression, and intestinal metabolic profiles have not yet been fully elucidated.

In this study, C57BL/6J mice were used as experimental animals, and hepatic fibrosis was induced by intraperitoneal injection of CCl_4_. The protective effects of two major bioactive components of *Spirulina platensis*, namely *Spirulina* polysaccharides (SPP) and phycocyanin (PC), against hepatic fibrosis were systematically evaluated. By integrating phenotypic assays (liver index, liver function parameters), hepatic histopathological analysis (H&E staining, Masson staining), molecular biological assays (mRNA expression of fibrosis- and intestinal barrier-related genes), and multi-omics techniques (16S rRNA gene sequencing, untargeted metabolomics), this study aims to clarify the protective effects and underlying mechanisms of SPP and PC against hepatic fibrosis, provide experimental evidence for the development of natural product-based interventions for hepatic fibrosis, and offer a new perspective for the high-value utilization of *Spirulina platensis* resources characteristic of Inner Mongolia.

## 2. Materials and Methods

### 2.1. Animals and Reagents

This study was approved by the Ethics Committee of Inner Mongolia Medical University [approval number: SYXK2025-0003] and was conducted in accordance with the Guidelines for the Care and Use of Laboratory Animals of Inner Mongolia Medical University. Thirty-two 5-week-old male C57BL/6J mice were purchased from Beijing Sibefu Biotechnology Co., Ltd., Beijing, China. The mice were housed under specific pathogen-free conditions with a temperature of 22–25 °C, relative humidity of 50–60%, and a 12 h light/12 h dark cycle, and were acclimatized for 1 week before the experiment. Carbon tetrachloride (CCl_4_) was purchased from Tianjin Fuchen Chemical Reagents Co., Ltd. (Tianjin, China). Animals were randomly assigned to experimental groups using a random number table by an investigator not involved in animal modeling or treatment administration. Investigators responsible for outcome assessment and data analysis were blinded to group allocation throughout the study. *Spirulina* polysaccharide (SPP) was prepared in our laboratory using a water extraction and ethanol precipitation method in previous work and stored in a desiccator until use. The raw *Spirulina* material was collected from the alkaline lake *Spirulina* platensis in the Mu Us Sandy Land, Ordos, Inner Mongolia (China). Phycocyanin (PC) was obtained from Kangsheng Algae Industry Co., Ltd., Etuoke Banner, Ordos, Inner Mongolia Autonomous Region, China. The mice were randomly divided into four groups with eight mice in each group: the normal control group (NC), model control group (MC), SPP-treated group (200 mg/kg/d SPP), and PC-treated group (200 mg/kg/d PC). SPP and PC were administered to the mice by intragastric gavage for 4 consecutive weeks. 

### 2.2. Establishment of a Murine Model of Hepatic Fibrosis

Thirty-two 5-week-old male C57BL/6J mice were acclimatized for 1 week and then randomly divided into four groups: the NC group, MC group, SPP group, and PC group (8 mice per group). All mice were fed a standard chow diet. The MC, SPP, and PC groups were intraperitoneally injected with a 28% CCl_4_ in olive oil at a dose of 2 mL/kg three times per week for 5 weeks, while the NC group was intraperitoneally injected with an equal volume of olive oil under the same conditions. One week after the start of modeling, the SPP and PC groups were orally administered SPP and PC solutions (0.2 mL, 200 mg/kg) once a day for 4 weeks, and the MC and NC groups were given an equal volume of distilled water.

### 2.3. Sample Collection

After 4 weeks of oral gavage, the mice were fasted for 4 h with free access to water, and their fasting body weights were recorded. Blood samples were collected by eyeball enucleation under anesthesia, and the whole blood was allowed to clot at room temperature for approximately 30 min. Serum was then separated by centrifugation at 3000 rpm for 15 min at 4 °C. After blood collection, the abdominal skin was disinfected with alcohol, and the abdominal cavity was opened through a U-shaped incision. The entire liver was excised, weighed, recorded, and photographed. The largest liver lobe was fixed in 4% paraformaldehyde for histopathological analysis, while the remaining liver tissues were placed in cryopreservation tubes, snap-frozen in liquid nitrogen, and stored in an ultra-low-temperature refrigerator at −80 °C. Fecal contents of the mice in each group were randomly collected, placed in cryopreservation tubes, snap-frozen in liquid nitrogen, and stored at −80 °C for multi-omics analysis. Serum biochemical parameters (ALT and AST), qPCR analyses of liver and intestinal tissues, and histopathological staining of liver tissues were performed independently by investigators who were not involved in group allocation or drug administration. All samples were labeled with anonymized codes (e.g., A, B), and the operators remained blinded to group identity throughout the experimental procedures. Fresh liver tissues were fixed in 4% paraformaldehyde for 48 h. The fixed tissues were rinsed with running water, dehydrated through a graded ethanol series, cleared in xylene, infiltrated with paraffin, and embedded in paraffin blocks. The embedded tissues were then sectioned into 3–4 μm-thick slices, mounted, baked, deparaffinized, and rehydrated. The sections were subjected to hematoxylin and eosin (H&E) staining and Masson’s trichrome staining according to standard protocols. After staining, the sections were dehydrated, cleared, mounted with neutral resin, and observed under a light microscope to evaluate hepatic histopathological changes. Microscopic images were acquired using a microscope system purchased from Chongqing Aote Optical Instrument Co., Ltd. (Chongqing, China).

### 2.4. Quantitative Real-Time PCR Analysis

Total mRNA was extracted from liver and colon tissues using TRIzol reagent (TransGen Biotech Co., Ltd., Beijing, China)and reverse-transcribed into cDNA using the Hifair^®^ III 1st Strand cDNA Synthesis Kit (TransGen Biotech Co., Ltd., Beijing, China). Quantitative real-time PCR was conducted using SYBR Green PCR Master Mix on a Fast Real-Time PCR System. Relative gene expression was calculated using the 2^−ΔΔCt^ method, normalized to GAPDH. The primer sequences used for quantitative real-time PCR are listed in Table 1.

### 2.5. 16S rRNA Gene Sequencing and Analysis

#### 2.5.1. Library Construction and Sequencing

The V3–V4 hypervariable region of the bacterial 16S rRNA gene was amplified using a proprietary primer set provided by GENEWIZ (Azenta Life Sciences, Burlington, MA, USA). PCR amplification was performed using genomic DNA extracted from fecal samples as the template. The PCR products were purified using magnetic beads, and sequencing libraries were prepared according to the manufacturer’s instructions. After quality assessment and accurate quantification, qualified libraries were pooled in equimolar amounts and subjected to paired-end sequencing on an Illumina sequencing platform. (Illumina, Inc., San Diego, CA, USA). The resulting raw reads were used for subsequent bioinformatic analysis of gut microbiota composition. The V3–V4 region of the 16S rRNA gene has limited taxonomic resolution, generally allowing reliable classification only at the genus level, while it is insufficient for accurate discrimination of closely related species or strains.

#### 2.5.2. Data Analysis

Raw sequencing data were processed by removing adapters and low-quality reads, followed by merging of paired-end reads. Chimeric sequences were subsequently removed to obtain high-quality effective sequences. Amplicon sequence variants (ASVs) were generated using a denoising algorithm, and representative ASV sequences were used for taxonomic annotation. The resulting taxonomic profiles were used to characterize the gut microbiota composition of each sample.

### 2.6. Untargeted Metabolomics Analysis

#### 2.6.1. Metabolite Extraction

Approximately 25 mg of fecal sample was weighed and mixed with 500 μL of pre-cooled extraction solution containing internal standards. The extraction solution consisted of methanol, acetonitrile, and water at a ratio of 2:2:1 (*v*/*v*/*v*) and was pre-cooled to −40 °C before use. Steel beads were added to the mixture, and the samples were homogenized at 35 Hz for 4 min, followed by sonication in an ice-water bath for 5 min. The homogenization–sonication procedure was repeated three times. The samples were then incubated at −40 °C for 1 h. After centrifugation, 300 μL of the supernatant was transferred to a 96-well filter plate. The filter plate and collection plate assembly was placed in a positive-pressure device (Waters Corporation, Milford, MA, USA), and the pressure was gradually increased to 6 psi for 3 min. The assembly was then removed from the device. Equal volumes of supernatant from all samples were pooled to prepare quality control (QC) samples.

#### 2.6.2. Instrumental Analysis

Polar metabolites were separated by ultra-performance liquid chromatography (UPLC) using a Vanquish UPLC system (Thermo Fisher Scientific, Waltham, MA, USA) equipped with a Waters ACQUITY UPLC BEH Amide column (Waters Corporation, Milford, MA, USA; 2.1 mm × 50 mm, 1.7 μm). Mobile phase A was an aqueous solution containing 25 mmol/L ammonium acetate and 25 mmol/L ammonia solution, and mobile phase B was acetonitrile. The autosampler temperature was set at 4 °C, and the injection volume was 2 μL. Mass spectral data were acquired in both positive and negative ion modes using an Orbitrap Exploris 120 mass spectrometer (Thermo Fisher Scientific) controlled by Xcalibur software version 4.4 (Thermo Fisher Scientific). Both MS1 and MS/MS data were collected for metabolite detection and structural annotation.

#### 2.6.3. Data Processing

Raw metabolomics data were converted to mzXML format using ProteoWizard software version 3.0.24054. Peak detection, alignment, and integration were performed using a custom R-based workflow. The analyses were performed using R version 3.6.3 with multiple packages including ggplot2 (3.3.5), pheatmap (1.0.12), ggpubr (0.4.0), and others. Metabolite annotation was conducted by matching retention time, accurate mass-to-charge ratio, and MS/MS spectra against an in-house metabolite database. The annotated metabolite data were then used for downstream statistical analysis and visualization using an in-house R package.

### 2.7. Statistical Analysis

All data were anonymized using group-specific codes, with group identifiers removed prior to analysis. Data analysis was performed by an independent investigator who was not involved in group allocation, animal handling, or experimental procedures. Group identities were only revealed after the completion of statistical analysis. All statistical analyses and data visualization were performed using GraphPad Prism 10.1.2 software. Comparisons between two independent groups were performed using an unpaired Student’s *t*-test. Comparisons among multiple groups were conducted using one-way analysis of variance (ANOVA), followed by Tukey’s post hoc test when appropriate. For microbiome and metabolomics analyses involving multiple comparisons, *p*-values were adjusted using the Benjamini–Hochberg false discovery rate (FDR) method. A *p*-value or adjusted *p*-value < 0.05 was considered statistically significant. Statistical significance was indicated as follows: * *p* < 0.05 and ** *p* < 0.01.

## 3. Results

### 3.1. SPP Ameliorates Hepatic Fibrosis in Mice

**Figure 1:**
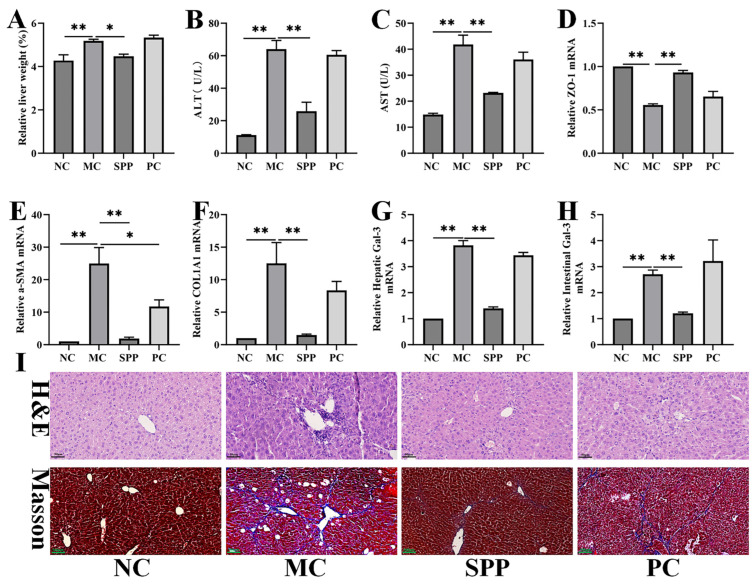
Liver index (liver weight/body weight) (**A**); mouse serum ALT (**B**); AST (**C**); relative mRNA expression of intestinal ZO-1 (**D**); relative mRNA expression of hepatic COL1A1 and α-SMA (**E**,**F**); relative mRNA expression of hepatic Gal-3 (**G**); relative mRNA expression of intestinal Gal-3 (**H**); H&E and Masson staining images of mouse liver tissue (**I**) ** indicates significance (*p* < 0.01); * indicates significance (*p* < 0.05). Scale bars represent 50 μm in H&E-stained images and 100 μm in Masson-stained images.

The general status of mice in each group was observed and recorded during the experiment. Mice in the NC group showed active behavior, shiny fur, formed feces, and clear urine; in contrast, mice in the MC group exhibited obvious abnormal manifestations including reduced activity, emaciation, rough fur, dark urine, and loose stools. Mice in the SPP group showed an improved general condition compared with the MC group, while the PC group had a similar general status to the MC group. After the experiment, the liver was excised and weighed to calculate the liver index (Figure 1A). Statistical analysis showed that SPP significantly reduced the liver weight and liver index of fibrotic mice, while PC had no such effect. Compared with the NC group, the serum levels of alanine aminotransferase (ALT) and aspartate aminotransferase (AST) were significantly increased in the MC group (Figure 1B,C), and SPP intervention effectively reversed these increases, whereas PC had no significant improvement effect. Collectively, these results indicate that SPP exerts a protective effect against CCl_4_-induced hepatic injury.

The relative mRNA expression of zonula occludens-1 (ZO-1) in the intestine was significantly decreased in the MC group compared with the NC group (Figure 1D), and SPP intervention upregulated the expression of ZO-1; PC slightly increased ZO-1 expression, but the difference was not statistically significant, suggesting that SPP can improve intestinal barrier-associated gene expression. The relative mRNA expressions of α-smooth muscle actin (α-SMA) and collagen type I alpha 1 chain (COL1A1) in the liver were markedly elevated in the MC group compared with the NC group (Figure 1E,F), and SPP treatment significantly downregulated their expressions; PC showed a slight decreasing trend with no statistical significance, indicating that SPP suppresses the transcriptional activation of fibrosis-related markers. The relative mRNA expression of galectin-3 (Gal-3) in both the liver and intestine was significantly increased in the MC group compared with the NC group (Figure 1G,H), and SPP intervention reduced Gal-3 expression in both tissues, while PC had no inhibitory effect, suggesting that SPP mitigates hepatic and intestinal inflammation.

H&E staining was used to observe the histopathological changes and inflammatory cell infiltration (Figure 1I): the NC group showed intact hepatic tissue structure, regular arrangement of hepatocytes, and no obvious inflammatory cell infiltration or hepatocellular injury; the MC group exhibited severe hepatic pathological changes, including disordered hepatocellular structure, inflammatory cell aggregation, and marked lymphocytic infiltration in the portal areas. Compared with the MC group, the SPP group showed a significant reduction in the degree of hepatic pathological damage, a regular arrangement of hepatocytes, and decreased inflammatory cell infiltration, while the PC group still had obvious hepatic pathological changes. Masson staining (collagen fibers stained blue) was performed to observe fibrous tissue distribution (Figure 1I): the NC group had almost no collagen fiber production, the MC group showed massive proliferation of hepatic collagen fibers, the SPP group had significantly reduced collagen deposition compared with the MC group, and the PC group still exhibited obvious collagen fiber deposition.

### 3.2. SPP Modulates the Gut Microbiota Composition in Mice with Hepatic Fibrosis

**Figure 2:**
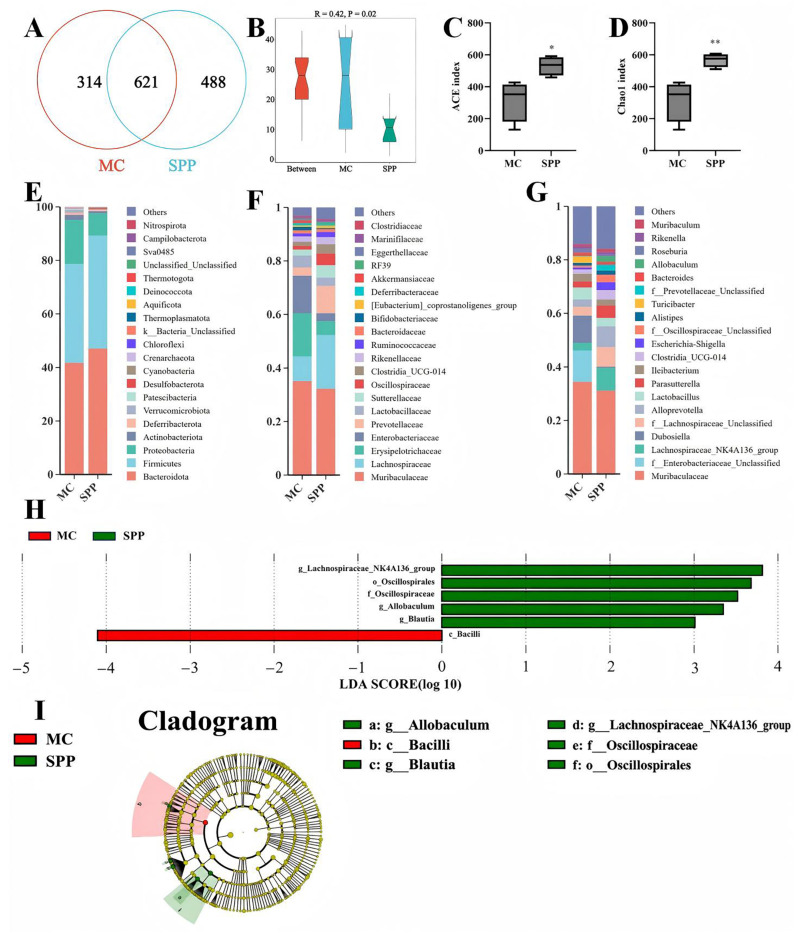
(**A**) Composition of gut microbes at genus level. (**B**) Anosim analysis of inter-group differences. (**C**) ACE index. (**D**) Chao1 index. (**E**) Differences in intestinal biota composition at phylum level. (**F**) Differences in intestinal biota composition at family level. (**G**) Differences in intestinal biota composition at genus level. (**H**) Linear discriminant analysis Effect Size (LEfSe) analysis identifying major biomarker taxa in the two groups (LDA score > 3.5). (**I**) Histogram of LDA scores. Yellow nodes represent taxa not significantly different between groups, serving as background in the cladogram. * *p* < 0.05; ** *p* < 0.01.

Through 16S rRNA sequencing analysis, Venn diagrams were constructed to identify unique or shared taxa among different sample groups, providing a visual representation of taxa composition similarity and overlap between sample groups. As shown in (Figure 2A), the MC and SPP groups shared 621 taxa, the MC group had 314 unique taxa, and the SPP group had 488 unique taxa. It was observed that after SPP treatment, the number of unique taxa in the SPP group mice significantly increased compared to the MC group, clearly indicating that dietary SPP altered the quantity of gut microbiota in mice, thereby modifying their original gut microbiota structure. Anosim analysis revealed significant inter-group differences (*p* < 0.05, R = 0.42), and the rank-based sorting results based on inter-sample distances were presented as boxplots (Figure 2B), further demonstrating significant differences between the two sample groups. Additionally, the α-diversity index in the MC group was relatively low, while the ACE and chao1 indices in the SPP-treated group significantly increased compared to the MC group (*p* < 0.05; Figure 2C,D), suggesting that SPP could enhance the taxa richness of gut microbiota in liver fibrosis mice. After SPP treatment in liver fibrosis mice, 16S sequencing was used to evaluate the microbial composition in the gut, with the *Firmicutes* and *Bacteroidetes* phyla being the most prevalent (Figure 2E). The *Firmicutes* phylum exhibited the highest relative abundance in the microbial community, accounting for 47.15%. At the family level, the top three microbial families were *unclassified_Muribaculaceae*, *Erysipelotrichaceae*, and *Helicobacteriaceae* (Figure 2F). At the genus level, the top three genera were *Muribaculaceae*, *Dubosella*, and *Alpenibacter* (Figure 2G). Linear Discriminant Analysis Effect Size (LEfSe) analysis (LDA score > 3.5) was performed to identify specific microbial taxa responsible for intergroup compositional differences. Consistent results from the phylogenetic tree and LDA bar plot indicated significant microbial community divergence (Figure 2H,I). *Bacilli* were significantly enriched in the MC group, while multiple *Firmicutes* taxa were markedly enriched in the SPP group, including *Lachnospiraceae_NK4A136_group*, *Oscillospirales*, *Oscillospiraceae*, *Allobaculum*, and *Blautia*. These taxa represent the may represent characteristic microbial features associated with SPP treatment.

### 3.3. SPP Regulates the Intestinal Metabolite Profile in Mice with Hepatic Fibrosis

**Figure 3:**
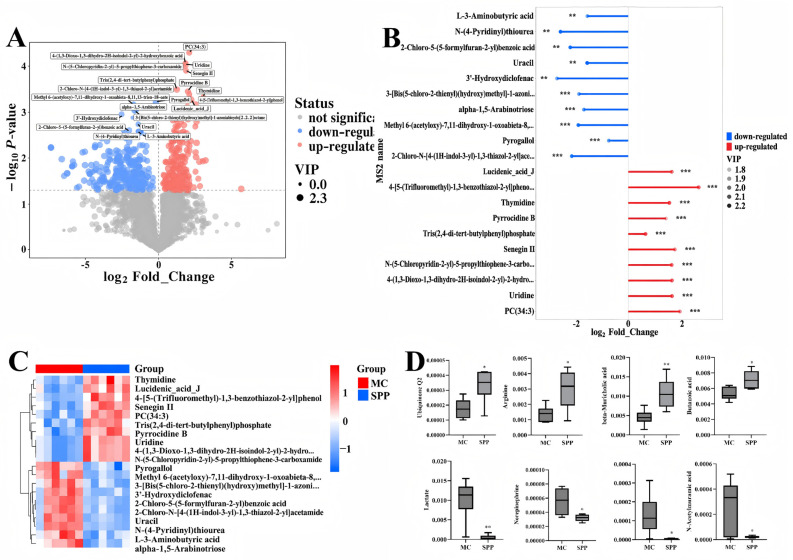
Effect of SPP on intestinal metabolites in mice with hepatic fibrosis. (**A**–**C**) Volcano plot, matchstick plot, and heatmap of differential metabolites. (**D**) Significantly altered differential metabolites. * *p* < 0.05; ** *p* < 0.01; *** *p* < 0.001.

Metabolomic analysis was performed using ultra-performance liquid chromatography UPLC-MS/MS to characterize fecal metabolic profiles. The screening criteria for differential metabolites were VIP > 1 and *p*-value < 0.05, resulting in the identification of 510 differential metabolites. Among these, 232 were up-regulated and 278 were down-regulated (Figure 3A–C). Subsequently, 69 core differential metabolites were identified. Compared with the MC group, the SPP group exhibited increased relative levels of arginine, butyrate, ubiquinone Q2, and β-ursolic acid, as well as significantly decreased levels of lactate, norepinephrine, *N*-acetylmuramic acid, and *N*-lactoylphenylalanine (Figure 3D). The current study demonstrates that increased production of butyrate by gut bacteria is associated with alleviation of hepatic fibrosis. These results suggest that SPP may influence the production of gut metabolites. This finding is consistent with previous experimental data, which indicated that multiple up-regulated bacterial groups can produce butyrate.

### 3.4. SPP Can Improve Liver Fibrosis by Regulating Multiple Metabolic Pathways

**Figure 4:**
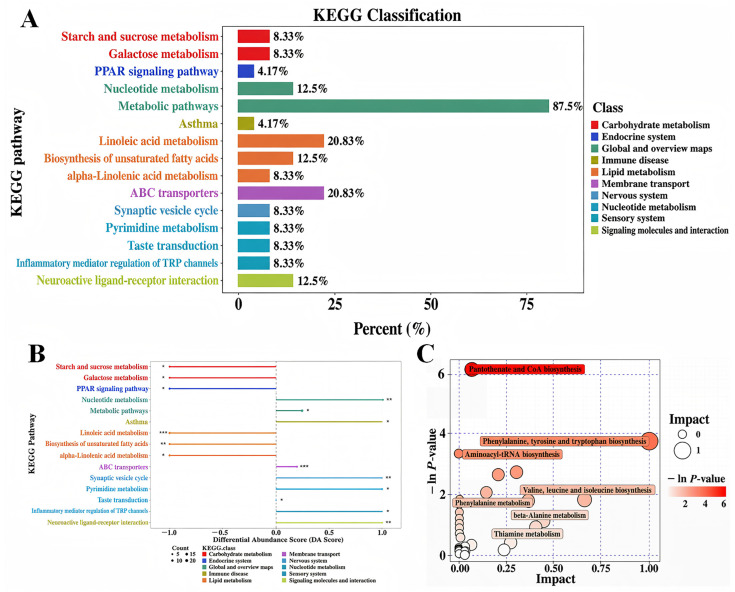
(**A**) KEGG classification of differential metabolites. (**B**) Differential abundance score plot. (**C**) Bubble plot of metabolic pathway enrichment analysis. * *p* < 0.05; ** *p* < 0.01; *** *p* < 0.001.

Kyoto Encyclopedia of Genes and Genomes (KEGG) enrichment analysis was performed on the differential metabolites to explore the metabolic pathways potentially affected by SPP treatment. The results showed that the differential metabolites were mainly enriched in pathways related to general metabolic pathways, biosynthesis of amino acids, and ABC transporters (Figure 4A). The most significantly upregulated pathways included arginine biosynthesis, phenylalanine metabolism, and arginine and proline metabolism, while digestive system-related pathways such as mineral absorption and protein digestion and absorption were downregulated (Figure 4B). The differential metabolites were also enriched in nucleotide metabolism, linoleic acid metabolism, biosynthesis of amino acids, and biosynthesis of pantothenate and CoA (Figure 4C). These findings suggest that SPP ameliorates hepatic fibrosis by regulating multiple metabolic pathways in the gut.

### 3.5. SPP Can Improve Liver Fibrosis by Regulating the Intestinal Flora and Related Metabolites

**Figure 5:**
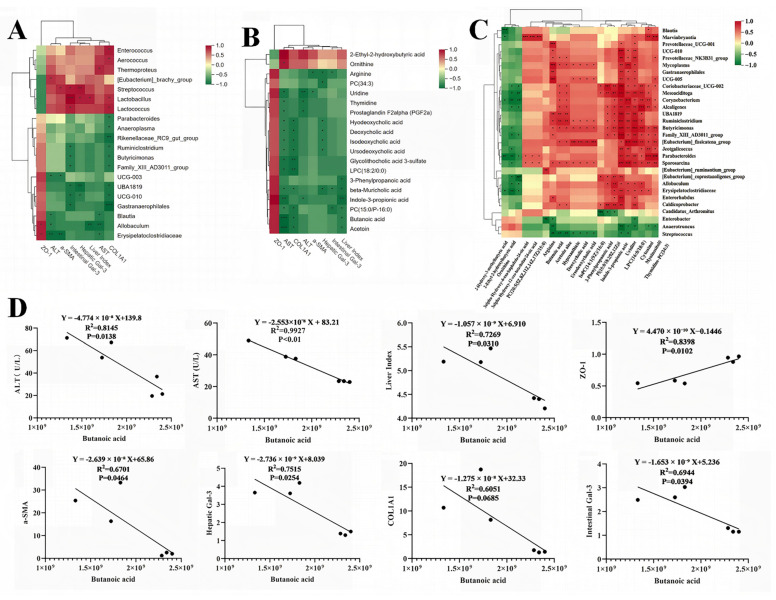
(**A**) Heatmap of potential correlations between bacterial genera and hepatic fibrosis-related indices. The *x*-axis represents hepatic fibrosis-related indices, and the *y*-axis represents bacterial genera. (**B**) Heatmap of potential correlations between intestinal differential metabolites and hepatic fibrosis-related indices. The *x*-axis represents hepatic fibrosis-related indices, and the *y*-axis represents intestinal differential metabolites. (**C**) Heatmap of potential correlations between bacterial genera and intestinal differential metabolites. The *x*-axis represents intestinal differential metabolites, and the *y*-axis represents bacterial genera. (**D**) Scatter plot of butyrate correlation with liver fibrosis-related indicators. The *x*-axis represents liver fibrosis-related indicators, and the *y*-axis represents butyrate. In the heatmaps, red indicates a positive correlation, green indicates a negative correlation, and deeper color represents stronger correlation. * *p* < 0.05; ** *p* < 0.01; *** *p* < 0.001.

Spearman rank correlation analysis was performed on bacterial genera with a relative abundance ≥ 1%. As shown in Figure 5A, after SPP treatment, *Enterococcus*, *Aerococcus*, *Streptococcus*, and other genera were positively correlated with AST, ALT, inflammatory markers, fibrosis-related markers, and liver index, while negatively correlated with intestinal tight junction-related markers. In contrast, *Parabacteroides*, *Rikenellaceae_RC9_gut_group*, *Ruminiclostridium*, and *Butyricimonas* were negatively correlated with AST, ALT, inflammatory markers, fibrosis-related markers, and liver index, while positively correlated with intestinal tight junction-related markers.

Spearman rank correlation analysis was also conducted between fecal metabolites and liver fibrosis-related indicators. As shown in Figure 5B,D, after SPP treatment, butyric acid was negatively correlated with AST, ALT, inflammatory markers, fibrosis-related markers, and liver index, while positively correlated with intestinal tight junction-related markers. In contrast, 2-ethyl-2-hydroxybutyric acid showed positive correlations with AST, ALT, inflammatory markers, fibrosis-related markers, and liver index, and negative correlations with intestinal tight junction-related markers.

Furthermore, Spearman correlation coefficients were used to analyze the relationships between gut microbiota and metabolites in SPP-treated hepatic fibrosis mice. *Butyricimonas* and *Ruminiclostridium* were positively correlated with butanoic acid and ursodeoxycholic acid, whereas *Streptococcus* and *Enterobacter* were negatively correlated with 2-ethyl-2-hydroxybutyric acid and 7-ketolithocholic acid (Figure 5C).

Overall, these results suggest that SPP may ameliorate hepatic fibrosis by modulating gut microbiota composition and associated metabolite profiles.

## 4. Discussion

A key pathological feature of hepatic fibrosis is the activation of HSCs and subsequent collagen deposition induced by hepatocellular injury, and abnormal liver function indices (elevated ALT and AST) and increased liver index are commonly associated with hepatic injury [9,10]. In this study, mice in the MC group exhibited obvious disordered hepatocellular structure, inflammatory infiltration, and collagen deposition, accompanied by a significant increase in ALT/AST levels and liver index (*p* < 0.001). These findings indicate that the CCl_4_-induced hepatic fibrosis model was successfully established and showed pathological characteristics consistent with liver injury and fibrotic progression.

SPP intervention effectively improved the above pathological changes: at the phenotypic level, the liver index of mice in the SPP group was significantly lower than that in the MC group (*p* < 0.05), and the serum levels of ALT and AST were significantly reduced (*p* < 0.001). These results suggest that SPP may alleviate CCl_4_-induced liver injury and improve liver function-related indicators. At the histopathological level, H&E staining showed improved regularity of hepatocellular arrangement and reduced inflammatory cell infiltration in the SPP group, and Masson staining further confirmed a decrease in collagen fiber deposition, suggesting that SPP may attenuate the core pathological process of hepatic fibrosis—excessive collagen synthesis and deposition. This effect is closely related to the free radical scavenging ability of SPP: previous studies have confirmed that SPP can reduce oxidative stress levels by activating the antioxidant enzyme system (SOD, GSH-Px) [11,12,13]. In this study, SPP may have reduced CCl_4_-induced oxidative damage in hepatocytes, thereby indirectly limiting hepatic stellate cell activation and fibrogenesis. However, further validation using oxidative stress markers and protein-level assessment of fibrosis-related pathways is needed to confirm this mechanism.

PC, another bioactive component of *Spirulina*, did not show a comparable protective effect under the experimental conditions used in this study. This difference may be related to the distinct structural characteristics, bioavailability, and biological targets of SPP and PC. Although PC has been reported to possess antioxidant activity [14], its proteinaceous structure differs substantially from the polysaccharide structure of SPP, which may partly explain their different effects on CCl_4_-induced hepatic fibrosis. In the present study, PC at the tested dose did not significantly improve liver injury markers, fibrosis-related gene expression, or histopathological changes. These findings suggest that SPP exerted more pronounced anti-fibrotic effects than PC in this model. However, further studies involving dose–response evaluation, PC stability assessment, and mechanistic validation are needed before excluding the potential anti-fibrotic activity of PC.

The gut microbiota can influence hepatic inflammation and fibrogenesis through microbial metabolites and gut-derived factors, such as short-chain fatty acids and lipopolysaccharide (LPS) [15]. In the present study, mice in the MC group exhibited gut microbiota dysbiosis, which may be associated with CCl_4_-induced hepatic injury and related alterations in intestinal function and bile acid metabolism.

16S rRNA gene sequencing showed that the MC and SPP groups shared 621 ASVs, whereas 314 and 488 ASVs were unique to the MC and SPP groups, respectively. ANOSIM analysis further revealed a significant difference in gut microbiota structure between the MC and SPP groups (R = 0.42, *p* = 0.02), suggesting that SPP treatment altered the gut microbial community composition in mice with hepatic fibrosis.

In terms of microbiota composition, the ratio of *Firmicutes* (relative abundance 47.15%) to *Bacteroidetes* in the SPP group tended to be balanced, and the abundance of beneficial bacterial genera such as *Allobaculum*, *Blautia*, and *Odoribacter* was significantly increased. Spearman correlation analysis further showed that these genera were negatively correlated with ALT, AST, liver index, and fibrotic factors (α-SMA, COL1A1), and positively correlated with the intestinal barrier-related marker ZO-1. These results suggest that SPP-enriched potentially beneficial bacterial genera may exert their anti-fibrotic effects through two main pathways: first, *Allobaculum* and *Blautia,* associated with short-chain fatty acid metabolism, including butyrate production through metabolism, which serves as the main energy source for intestinal epithelial cells and promotes intestinal barrier repair [16]; second, *Odoribacter* reduces the level of intestinal LPS, thereby decreasing the inflammatory response in the liver induced by LPS entering the liver via the portal vein and inhibiting HSC activation [17]. However, because serum LPS levels, intestinal permeability, and absolute short-chain fatty acid concentrations were not directly measured in this study, these mechanisms require further experimental validation.

After SPP intervention, the relative abundance of several bacterial genera positively correlated with hepatic injury-related indicators, such as *Marvinbryantia* and *Lactobacillus*, was decreased. This finding suggests that SPP may selectively modulate gut microbiota composition by reducing taxa associated with liver injury and increasing taxa associated with improved intestinal barrier function and reduced fibrosis-related markers. However, because these results are based on correlation analysis, further functional validation is required to determine whether these bacterial changes directly contribute to the anti-fibrotic effects of SPP.

Increased intestinal permeability caused by intestinal barrier damage is a key link in “gut-derived hepatic injury”: intestinal LPS and bacterial metabolites can enter the portal vein through the damaged intestinal barrier, continuously activating hepatic inflammatory responses and accelerating fibrogenesis. The mRNA expression of the intestinal barrier-related marker was significantly decreased in the colonic tissue of mice in the MC group, while SPP intervention significantly upregulated ZO-1 expression and reduced the expression of the intestinal inflammatory factor Gal-3. Gal-3, an inflammation-associated lectin, can not only directly promote HSC activation but also increase intestinal permeability by destroying the tight junctions between intestinal epithelial cells [18,19,20]. The inhibitory effect of SPP on Gal-3 not only alleviates its direct damage to the liver but also may exert protective effects partly through modulation of the gut–liver axis of “gut-derived toxins-hepatic inflammation” by maintaining the intestinal barrier. This “bidirectional regulatory” effect further illustrates the unique value of SPP in the regulation of the gut-liver axis. In the PC group, no significant changes in ZO-1 and Gal-3 were observed, indicating that PC may have a weaker effect than SPP under the experimental conditions used in this study, which is an important reason for its poor anti-fibrotic effect.

Fecal metabolites, as important signaling molecules involved in the gut–liver axis, are closely associated with the development and progression of hepatic fibrosis. In the present study, untargeted metabolomics analysis showed that, compared with the MC group, the SPP group exhibited increased relative levels of arginine, butyrate, ubiquinone Q2, and β-muricholic acid, whereas the relative levels of lactate, norepinephrine, *N*-acetylmuramic acid, and *N*-lactoylphenylalanine were decreased.

The increased butyrate-related metabolic signal may be associated with the enrichment of bacterial genera with potential short-chain fatty acid-producing capacity, such as Allobaculum, as observed in the 16S rRNA sequencing results. Butyrate is an important energy source for intestinal epithelial cells and has been reported to contribute to intestinal barrier maintenance and the regulation of inflammatory responses through receptors such as GPR41 and GPR43 [21]. Therefore, SPP-associated changes in butyrate metabolism may partly contribute to the improvement of intestinal barrier-related and hepatic fibrosis-related indicators. However, targeted quantification of short-chain fatty acids is required to confirm changes in absolute butyrate levels.

Arginine is a precursor of nitric oxide (NO), which is involved in the regulation of hepatic microcirculation, inflammation, and fibrogenesis. The increased relative level of arginine observed after SPP treatment may therefore be associated with modulation of the arginine–NO metabolic axis. However, because NO levels and related enzymatic activities were not directly measured in this study, this mechanism requires further validation [22,23].

In addition, β-muricholic acid, a bile acid metabolite, was significantly increased in the SPP group. As bile acids are important signaling molecules regulating the farnesoid X receptor (FXR) pathway, the observed change in β-muricholic acid may suggest a potential involvement of bile acid–FXR signaling in the protective effects of SPP. Nevertheless, further targeted bile acid profiling and functional experiments are needed to confirm this possibility [24,25].

In terms of metabolic pathways, the differential metabolites associated with SPP treatment were mainly enriched in sphingolipid metabolism, linoleic acid metabolism, α-linolenic acid metabolism, and the biosynthesis pathways of branched-chain amino acids (valine, leucine, and isoleucine). Dysregulation of sphingolipid metabolism has been reported to contribute to alterations in hepatocyte membrane structure and hepatic stellate cell activation, suggesting that modulation of this pathway may be involved in the protective effects of SPP [26,27].

Linoleic acid and α-linolenic acid, as polyunsaturated fatty acids, can be metabolized into bioactive lipid mediators, including prostaglandins, which have been implicated in the regulation of inflammatory responses [28,29]. Therefore, SPP-associated alterations in these pathways may contribute to the regulation of hepatic inflammatory status.

In addition, abnormal branched-chain amino acid metabolism, such as elevated valine levels, has been associated with hepatic insulin resistance and lipid accumulation. The observed changes in branched-chain amino acid biosynthesis pathways in the SPP group may therefore reflect an improved hepatic metabolic environment, which could indirectly contribute to the attenuation of fibrogenesis [30]. However, these associations require further targeted metabolic and functional validation.

In summary, SPP alleviates CCl_4_-induced hepatic fibrosis in mice, which may be associated with the modulation of gut microbiota composition and the regulation of host metabolic profiles. Specifically, SPP treatment was linked to alterations in key bacterial taxa and fecal metabolites, suggesting a potential role in gut–liver axis regulation.

This study has several limitations. For instance, gut microbiota analysis was performed mainly at the genus level based on 16S rRNA sequencing, which limits resolution at the species and functional levels. In future studies, higher-resolution sequencing approaches combined with functional metagenomic or metabolomic analyses will be employed to further elucidate microbial functions. A limitation of the present study is that protein-level validation of key genes was not performed. Although the mRNA expression levels of fibrosis- and intestinal barrier-related markers (such as α-SMA, COL1A1, ZO-1, and Gal-3) provided important insights into the transcriptional regulation associated with SPP treatment, mRNA abundance does not always directly reflect corresponding protein expression, particularly in complex pathological conditions such as hepatic fibrosis.

Future studies should incorporate protein-level analyses to further validate the observed gene expression changes and strengthen the mechanistic understanding of the protective effects of SPP against hepatic fibrosis. In addition, validation experiments with larger sample sizes and targeted functional assays are needed to confirm the observed associations and underlying mechanisms.

The primary objective of this study was to investigate whether *Spirulina* polysaccharide (SPP) could attenuate or reverse CCl_4_-induced hepatic fibrosis in mice. Accordingly, the gut microbiota and fecal metabolomic profiles were primarily compared between the model control group (MC) and the SPP-treated group (SPP), with a focus on identifying SPP-associated regulatory effects under pathological conditions.

However, the absence of a normal control (NC) group in the microbiota and metabolomics comparisons limits the ability to determine whether SPP restores the gut microbial and metabolic profiles to a physiological baseline or induces a distinct microbial and metabolic state beyond normal conditions. Therefore, the observed effects can only be interpreted as significant improvements relative to the diseased state (MC group), rather than full normalization.

Future studies incorporating a complete experimental design including NC–MC–SPP comparisons are necessary to comprehensively evaluate the extent to which SPP restores gut–liver axis homeostasis and to further clarify its mechanistic role in hepatic fibrosis.

## 5. Conclusions

SPP significantly alleviates CCl_4_-induced hepatic fibrosis in mice. The protective effects are associated with improved liver function, reduced collagen deposition, and attenuation of inflammatory responses. Mechanistically, SPP modulates gut microbiota composition and regulates key metabolic pathways, including short-chain fatty acid-related metabolism, thereby contributing to the restoration of gut–liver axis homeostasis. These findings suggest that SPP may serve as a potential natural therapeutic candidate for hepatic fibrosis.

## Figures and Tables

**Table 1:** Real-time quantitative PCR primers.

Gene	Forward Primer Sequence (5′–3′)	Reverse Primer Sequence (5′–3′)
GAPDH(Mouse)	AGCCAAAAGGGTCATCATCT	GGGGCCATCAGTCTTCT
Gal-3 (Mouse)	GGAGAGGGAATGATGTTGCCT	TCCTGCTTCGTGTTACACACA
COL1A1 (Mouse)	TAGGCCATTGTGTATGCAGC	ACATGTTCAGCTTTGTGGACC
α-SAM (Mouse)	TCGGATACTTCAGCGTCAGGA	AGCCTCCGACTTGTGAAGTG
ZO-1 (Mouse)	TGAACGTCCCTGACCTTTCG	GCTCTGAACGTTGGTCAGGA

## Data Availability

The original contributions presented in this study are included in the article. Further inquiries can be directed to the corresponding authors.
